# The Contribution of Genetic Variants of the Peroxisome Proliferator-Activated Receptor-Alpha Gene to High-Altitude Hypoxia Adaptation in Sherpa Highlanders

**DOI:** 10.1089/ham.2018.0052

**Published:** 2023-09-12

**Authors:** Fumiya Kinota, Yunden Droma, Nobumitsu Kobayashi, Toshimichi Horiuchi, Yoshiaki Kitaguchi, Masanori Yasuo, Masao Ota, Masayuki Hanaoka

**Affiliations:** ^1^ The First Department of Medicine, Shinshu University School of Medicine, Matsumoto, Japan.; ^2^ Division of Hepatology and Gastroenterology, Department of Medicine, Shinshu University School of Medicine, Matsumoto, Japan.

**Keywords:** high altitude, metabolism, peroxisome proliferator-activated receptor-alpha gene, Sherpas, single-nucleotide polymorphisms

## Abstract

Kinota, Fumiya, Yunden Droma, Nobumitsu Kobayashi, Toshimichi Horiuchi, Yoshiaki Kitaguchi, Masanori Yasuo, Masao Ota, and Masayuki Hanaoka. The contribution of genetic variants of the gene encoding peroxisome proliferator-activated receptor-alpha gene (*PPARA*) to high-altitude hypoxia adaptation in Sherpa highlanders. *High Alt Med Biol*. 24:186–192, 2023.—Sherpa highlanders, who play invaluable roles in the exploration of Mount Everest, have exceptional tolerance to hypobaric hypoxia. Sherpa people are well known to possess the traits determined by genetic background for high-altitude adaptation. The metabolic adaptation mechanism is one of the biological ways for Sherpa highlanders in protecting them from hypoxia stress at high altitude. Studies have suggested that the gene encoding *PPARA* is associated with metabolic adaptation in the Himalayan population of Tibetans. This study attempts to investigate the genetic variants of the *PPARA* in Sherpa highlanders and the association with high-altitude hypoxia adaptation. Seven single-nucleotide polymorphisms (SNPs; rs135547, rs5769178, rs881740, rs4253712, rs5766741, and rs5767700 in introns and rs1800234 in exon 6) in the *PPARA* were genotyped in 105 Sherpa highlanders who lived in the Khumbu region (3440 m above sea level) and 111 non-Sherpa lowlanders who resided in Kathmandu (1300 m) in Nepal. By means of analyses of genetic distances, genotype distributions, allele frequencies, linkage disequilibrium, and haplotype constructions of the seven SNPs in the Sherpa highlanders versus the non-Sherpa lowlanders, it was revealed that the frequencies of minor alleles of rs4253712, rs5766741, rs5767700, and rs1800234 SNPs, as well as the frequency of haplotype constructed by the minor alleles of rs5766741–rs5767700–rs1800234, were significantly overrepresented in the Sherpa highlanders in comparison with the non-Sherpa lowlanders. The results strongly suggest that the genetic variants of the *PPARA* are likely to contribute to the high-altitude adaptation in Sherpa highlanders.

## Introduction

Sherpa highlanders, who play invaluable roles in the exploration of Mount Everest, have exceptional tolerance to hypobaric hypoxia. Sherpas are renowned in the international climbing and mountaineering community for their hardiness, expertise, and experience at very high altitudes; indeed, these qualities may be because of natural selection, as their ancestors have lived and reproduced at high altitude for hundreds of generations, with hypoxia exerting constant evolutionary pressure (Gilbert-Kawai et al., 2014). Sherpa people are well known to possess the traits for high-altitude adaptation (Hochachka et al., 1996a, 1996b; Horscroft et al., 2017). Physiological systems such as the respiratory system, cardiac vasculature, and hematology are adaptive to high-altitude hypoxia, with Sherpas showing phenotypes of increased lung diffusing capacity, elevated levels of bioactive nitric oxide products and other biovasodilators, and unelevated hemoglobin levels that sustain physiological functions at sea level rates under conditions of hypobaric hypoxia (Hanaoka et al., 2012; Gilbert-Kawai et al., 2014). Of note, the metabolic defenses of Sherpas against hypobaric hypoxia efficiently protect their hearts and brains from hypoxic stress at high altitudes (Morpurgo et al., 1976; Holden et al., 1995; Hochachka et al., 1996a, 1996b). The heart and brain are two organs of the human body that has the highest oxygen demands and the lowest rates of ATP production and utilization (Stanley et al., 2005; Du et al., 2008).

A genome-wide study has suggested that the gene encoding peroxisome proliferator-activated receptor-alpha (*PPARA*) exhibits a signal of natural selection for high-altitude adaptation in indigenous Tibetans living on Tibet Plateau (Simonson et al., 2010). Peroxisome proliferator-activated receptors (PPARs) are transcription factors that belong to the nuclear hormone receptor superfamily and regulate the expressions of several genes involving in metabolic processes (Dreyer et al., 1993). The PPAR-α subtype can be activated by certain natural and synthetic ligands (such as polyunsaturated fatty acids) and then modulates DNA transcription by binding to specific nucleotide sequences located in the regulatory regions of target genes (Contreras et al., 2013). The activation of PPAR-α by its ligands can modify multiple biological processes in the cell that are important in the mechanism associated with metabolism (Dreyer et al., 1993; Contreras et al., 2013).

The studies by Ge et al. (2012, 2015) demonstrated that the metabolic adaptation was associated with the *PPARA* haplotypes for mechanisms of high-altitude adaptation in Tibetans. Of note, a recent study revealed that the putatively advantageous alleles in metabolic adaptation to hypobaric hypoxia were enriched in Sherpa highlanders for certain single-nucleotide polymorphisms (SNPs) in the *PPARA*, and the enrichment was associated with the adaptive phenotypes in Sherpa highlanders (e.g., lower capacity for fatty acid oxidation in skeletal muscle, enhanced efficiency of oxygen utilization, and improved muscle energetics) in protecting them against oxidative stress (Horscroft et al., 2017).

We genotyped seven SNPs in the *PPARA* (rs135547, rs5769178, rs881740, rs4253712, rs5766741, and rs5767700 in introns and rs1800234 in exon 6) in 105 Sherpa highlanders permanently living at 3440 m above sea level and 111 non-Sherpa lowlanders living in Kathmandu at 1300 m. The genetic distances, genotype distributions, allele frequencies, linkage disequilibrium, and haplotype constructions were analyzed regarding the variances of the seven SNPs in the *PPARA* between the highlanders and lowlanders.

## Materials and Methods

### Ethics statement

The study protocol was developed in accordance with the principles outlined in the Declaration of Helsinki of the World Medical Association (World Medical Association, 2013) and was approved by the Ethics Committee of Shinshu University (Matsumoto, Japan) and the Nepal Health Research Council (Kathmandu, Nepal). The protocol was explained individually to each Sherpa highlander and non-Sherpa lowlander, and informed consent written in Nepalese was obtained by signature or by fingerprint if the subject was illiterate.

### Study populations

#### Sherpa highlanders

This group comprised 105 Sherpas who lived in Namche Bazaar village (3440 m) in the Khumbu region of Nepal (Table 1). Namche Bazaar is one of the most popular trekking junctions in proximity to Mt. Everest and other famous mountain peaks. Geographically, it is ∼200 km from Kathmandu, the capital of Nepal. The Sherpas voluntarily participated in this investigation. The Sherpa clan was identified with the Sherpa surname and confirmed by a senior native Sherpa. All enrolled Sherpas were born and permanently resided in Namche Bazaar and they had no history of intermarriage with other ethnic groups. The information about demography, health status, altitude residence, and mountaineering history was obtained by interview. The medical interviews and physical examinations excluded chronic mountain sicknesses (CMS) and other cardiopulmonary disorders. The percutaneous arterial oxygen saturation (SpO_2_) and heart rate were measured using a pulse oximeter (Pulsox-3; Minolta, Osaka, Japan) with a probe connecting to a finger. Venous blood samples were also taken and put into tubes containing anticoagulant ethylenediaminetetraacetic acid for DNA extraction.

**Table 1.** Phenotypes of Sherpa Highlanders and Non-Sherpa Lowlanders

Phenotypes	Sherpa highlanders at 3440 m	Non-Sherpa lowlanders at 1300 m	*p*
No. of subjects	105	111	
Male:female	44:61	53:58	0.388^a^
Age, years	31.2 ± 0.8	29.9 ± 0.8	0.251^b^
Dwelling at respective altitude, years	30.8 ± 0.8	20.7 ± 1.0	<0.0001^b^
Trekking guides and porters, *n* (%)	33 (31.4)	None	0.0001^a^
Mean altitude exposed, m	5701.4 ± 119.1	2688.6 ± 150.4	< 0.0001^b^
Highest altitude reached, m	8850	5300	
Oxygen saturation, %	93.3 ± 0.2	96.6 ± 0.2	< 0.0001^b^
Heart rate, bpm	80.7 ± 1.1	86.8 ± 1.5	0.0013^b^

Continuous data are expressed as mean ± SEM.

^a^ Compared by contingency table (2 × 2), df = 1.

^b^ Compared by unpaired *t*-test.

bpm, beats per minute; SEM, standard error of the mean.

The 105 Sherpa highlanders were composed of 44 men and 61 women. They had dwelt at Namche Bazaar for 30.8 ± 0.8 years, almost equal to their average age of 31.2 ± 0.8 years, suggesting that they were permanent residents in Namche Bazaar. None of the Sherpa highlanders complained of symptoms of CMS. Thirty-three of the 105 (31.4%) Sherpa highlanders were trekking guides and porters in the Himalayan region. Among them, 13 Sherpa men experienced the expeditions to mountains over 8000 m and 6 of the 13 Sherpas have reached the summit of Mt. Everest. The average of SpO_2_ was 93.3% ± 0.2% and the heart rate was 80.7 ± 1.1 beats per minute in the Sherpa highlanders at 3440 m.

#### Non-Sherpa lowlanders

This group comprised 111 non-Sherpa lowlanders who lived in Kathmandu (1300 m) in Nepal (Table 1). The protocol for the recruitment of subjects and collection of blood samples were followed as that in Namche Bazaar. In history, the non-Sherpa lowlanders in Nepal have shared politics, economics, and cultures with Sherpa highlanders for the past five centuries (Sherpa, 2008), which provides considerable equivalent environments regarding society and medical health services for both the populations. The high-altitude exposure was different between the two study populations (Table 1).

### Single-nucleotide polymorphisms

The *PPARA* is located on chromosome 22q13.31 (22: 46,150,553–46,243,755); it spans 83.7 kb and contains eight exons (Vohl et al., 2000). The selection criteria for the SNPs genotyped in this study were based on the following: (1) 1000 Genomes Project data that were accessed using the Ensembl GRCH37; (2) the Human Genetic Variation Database (HGVD) (www.hgvd.genome.med.kyoto-u.ac.jp/index.html); and (3) the Applied Biosystems SNP genotyping database. We specifically looked for the following: (1) SNPs located within the *PPARA* gene; (2) density of at least 1 SNP per 10 kb; (3) genetic distances of the fixation index (*F*_ST_) for the SNPs between South and East Asians were <0.05 (calculated from the 1000 Genomes Project data; Supplementary Table S1; Supplementary Data are available online at www.liebertpub.com/ham); (4) preference to SNPs of the expression of quantitative trait loci (eQTL); and (5) availability of Applied Biosystems validation assays.

The *F*_ST_ is a measure of population differentiation because of genetic structure. An *F*_ST_ value of zero indicates no divergence between populations, and a value of 1 indicates complete genetic variation between populations because of population stratification (Holsinger and Weir, 2009). It is estimated that the *F*_ST_ is 0.12 across the autosomes of global human populations (Lewontin, 1972; Duan et al., 2008). The genomic loci of eQTL contribute to variations in the expression levels of mRNA (Battle and Montgomery, 2014). The eQTL information was obtained through the HGVD that was a central data resource of Asian genetic variations and associations between the variation and the transcription levels of genes (Narahara et al., 2014; Higasa et al., 2016).

Using these selection criteria, we identified seven SNPs for the *PPARA* in the present genotyping: rs135547, rs5769178, rs881740, rs4253712, rs5766741, and rs5767700 in introns and rs1800234 in exon 6. Of these, the rs881740, rs4253712, and rs5766741 SNPs are eQTL loci, and rs1800234 is a nonsynonymous substitution (V227A) in the *PPARA*.

### Genotyping

The genomic DNA samples were extracted from the cells in venous blood of all subjects by phenol extraction as described previously (Droma et al., 2006). The SNP Genotyping Assay Mix contained the forward and reverse primers, the FAM™ and VIC™ dyes, and minor groove binder-labeled probes for the seven SNPs (Applied Biosystems, Foster City, CA). Allele discrimination was performed using the TaqMan^®^ SNP Genotyping Assay with the Applied Biosystems 7500 Fast Real-Time PCR System (Applied Biosystems, Inc.) following the manufacturer's instructions. After thermal cycling, genotype data were automatically acquired and analyzed using sequence detection software (SDS v1.3.1; Applied Biosystems, Inc.)

### Statistical analysis

Continuous data are expressed as mean ± standard error of the mean. The differences in the categorical data between the two populations were analyzed by contingency table (2 × 2). Allele frequencies were calculated by allele counting and were expressed as decimals. The Hardy–Weinberg equilibrium (HWE) was calculated individually for each SNP in the two study groups using the Genepop software package (Guo and Thompson, 1992). The differences in the SNP variations between the two study groups were examined by the chi-square test (2 × 2 contingency table), as were the inherited effects of the minor alleles assuming dominant mode or recessive mode in the population of Sherpa highlanders. The strengths of the minor alleles in terms of the frequencies in Sherpa highlanders were estimated by odds ratio (OR) with the approximate 95% confidence interval (CI). The samples size of this study was necessary to find the OR of 3.0 statistical significance (two-sided test) in a case–control study for a power of 80%. The *p* values were corrected for multiple hypothesis tests with Bonferroni's method. A corrected *p* (*pc*) value of <0.05 was considered significant.

The linkage disequilibrium (LD) of the seven SNPs was examined with Haploview 4.2 software to derive the pairwise LD measurements of the *D*′ value (Barrett et al., 2005). The logarithm of odds (LOD) provides a confidence measure of the *D*′ value. The genetic block structures were generated using the Haploview 4.2 software based on the default algorithm to generate strong LD of the SNPs using the common block definition (Gabriel et al., 2002). The frequencies of haplotypes in the genetic blocks were estimated based on the maximum-likelihood values by using the Haploview 4.2 software. Significant differences in the haplotype frequencies between the two study groups were examined by the chi-square test. The OR with approximate 95% CI was calculated. A *p* value of <0.05 was considered significant.

## Results

### The genetic distances of the seven SNPs in the *PPARA* in Sherpa highlanders versus non-Sherpa lowlanders

The genotype distributions and allele frequencies for each of the seven SNPs in the *PPARA* were in HWE for the two study groups. The genetic distances between the Sherpa highlanders and non-Sherpa lowlanders were in a range from 0.0103 to 0.0883 for the seven SNPs by the unbiased *F*_ST_ measurement (Supplementary Table S2). This indicated that the population stratification of the Sherpa highlanders and non-Sherpa lowlanders was minor for these seven SNPs according to the *F*_ST_ for global human populations (Lewontin, 1972; Duan et al., 2008).

### Genotype distributions and allele frequencies of the seven SNPs in the *PPARA* in Sherpa highlanders versus non-Sherpa lowlanders

Based on the minor population stratification of the seven SNPs between Sherpa highlanders and non-Sherpa lowlanders, we compared the genotype distributions and allele frequencies of these SNPs between the two populations. The results showed that of the seven SNPs, five SNPs (rs135547, rs4253712, rs5766741, rs5767700, and rs1800234) were significantly different in the genotype distributions and allele frequencies between the two study groups (Table 2). The frequencies of the minor alleles of rs4253712G, rs5766741C, rs5767700C, and rs1800234C were significantly higher in the Sherpa highlanders than non-Sherpa lowlanders (respectively, *pc* = 0.0329, 0.0357, 0.0126, and 0.0112; OR = 2.13, 2.01, 2.14, and 3.74). Particular attention was paid to rs4253712 and rs5766741 that are eQTL loci, and to rs1800234 that is a nonsynonymous substitution in the *PPARA*. In addition, the minor alleles of rs4253712G, rs5766741C, rs5767700C, and rs1800234C were assumed to follow a dominant mode (11 + 12/22) of inheritance in Sherpa highlanders (Table 2). On the contrary, the frequency of the major allele of rs135547C was significantly higher in the Sherpa highlanders than non-Sherpa lowlanders (*pc* = 0.0056; Table 2).

**Table 2.** Genotype Distribution and Allele Frequencies of the Seven Single-Nucleotide Polymorphisms in the Gene Encodes Peroxisome Proliferator-Activated Receptor-Alpha in the Sherpa Highlanders Versus Non-Sherpa Lowlanders

SNPs	Sherpa highlanders (*n* = 105)				Non-Sherpa lowlanders (*n* = 111)				*p*	*pc*	OR (95% CI)	*p*	*p*
	Genotype (freq)			Allele (freq)	Genotype (freq)			Allele (freq)					
	11	12	22	1	11	12	22	1	(1/2)	(1/2)	(1/2)	(11/12 + 22)*^a^*	(11 + 12/22)*^b^*
**rs135547 (G/C)**	0	0.21	0.78	0.11	0.04	0.39	0.57	0.23	**0.0008**	**0.0056**	0.41 (0.24–0.70)	0.0485	**0.0011**
rs5769178 (C/A)	0.04	0.25	0.71	0.16	0.04	0.16	0.80	0.11	0.1459	1.0213	1.51 (0.86–2.63)	0.9465	0.1023
rs881740 (G/A)^c^	0.04	0.25	0.71	0.16	0.05	0.15	0.80	0.11	0.1909	1.3363	1.44 (0.83–2.50)	0.7876	0.1024
**rs4253712 (G/A)** ^c^	0.04	0.35	0.61	0.21	0.03	0.17	0.80	0.10	**0.0047**	**0.0329**	**2.13 (1.25–3.62)**	0.6549	**0.0022**
**rs5766741 (C/T)** ^c^	0.04	0.42	0.54	0.25	0.04	0.21	0.75	0.13	**0.0051**	**0.0357**	**2.01 (1.23–3.28)**	0.9465	**0.0011**
**rs5767700 (C/T)**	0.07	0.40	0.53	0.27	0.04	0.21	0.75	0.14	**0.0018**	**0.0126**	**2.14 (1.32–3.47)**	0.3134	**0.0012**
**rs1800234 (C/T)** ^d^	0	0.22	0.78	0.11	0	0.06	0.94	0.02	**0.0016**	**0.0112**	**3.74 (1.57–8.92)**	—	**0.0010**

Bold indicates data that are significantly different between the two populations. 1 is for minor allele and 2 is for major allele, and the SNP variant is shown as the rs number (minor allele/major allele).

^a^ 2 × 2 contingency table assuming the recessive mode with minor allele (11/12 + 22) of inheritance in Sherpa highlanders.

^b^ 2 × 2 contingency table assuming the dominant mode with minor allele (11 + 12/22) of inheritance in Sherpa highlanders.

^c^ eQTLs, the genomic loci that contribute to variations in the mRNA expression levels of the *PPARA*.

^d^ The nonsynonymous substitution V227A in the PPARA receptor.

The *p* values were calculated using the chi-square test (2 × 2 contingency table). The corrected *p* value (*pc*) was multiplied by the total number of the observed SNPs.

95% CI, 95% confidence interval; eQTL, expression quantitative trait loci; freq, frequency; OR, odds ratio; *pc*, corrected *p* value; *PPARA*, the gene encoding peroxisome proliferator-activated receptor-alpha; SNP, single-nucleotide polymorphism.

### Pairwise LD and the haplotypes of the seven SNPs in the *PPARA* in the Sherpa highlanders and non-Sherpa lowlanders

**FIG. 1.**
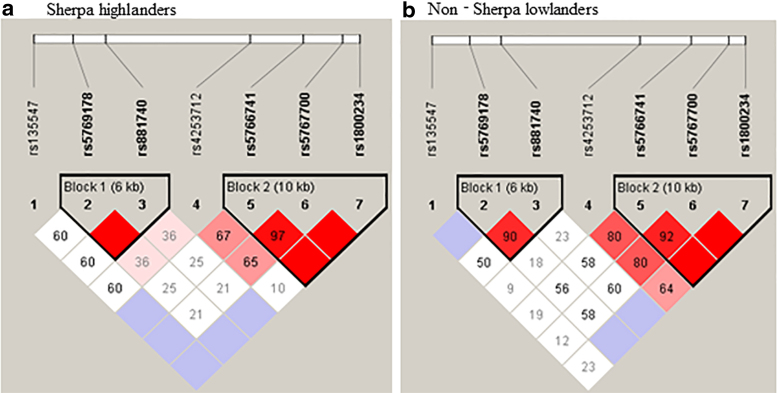
Pairwise linkage disequilibrium of the seven SNPs in the *PPARA* gene in Sherpa highlanders and non-Sherpa lowlanders. **(a)** Sherpa highlanders and **(b)** non-Sherpa lowlanders. *D*′ values are represented as diamonds. *Bright red*, *D*′ > 80 with LOD ≥3 indicating strong inherited linkage. *Light red/pink*, *D*′ ≤ 80 with LOD ≥ 3; *Blue*: *D*′ >80 with LOD <3; *White*: *D*′ ≤ 80 with LOD <3. LOD, logarithm of the odds; SNP, single-nucleotide polymorphism.

Pairwise LD analysis detected two haplotype blocks for the seven SNPs in the two study groups (Fig. 1). A *D*′ value of 100 and LOD >3 indicate that two loci are close to each other on a chromosome and are therefore likely to be inherited together (bright red in Fig. 1). Block 2 included rs5766741, rs5767700, and rs1800234 of three SNPs that covered 10 kb length of the gene (Fig. 1). Note that the rs5766741 is an eQTL locus and rs1800234 is a nonsynonymous substitution in the *PPARA*. The block 2 contained four types of haplotype and two of them were significantly different in frequencies between the two study groups (Table 3). Specifically, the frequency of haplotype C-C-C, which included the minor alleles of rs5766741, rs5767700, and rs1800234, was significantly higher in Sherpa highlanders (0.11) than non-Sherpa lowlanders (0.03, *p* = 0.0016) with a strong OR (3.74, 95% CI = 1.57–8.92) (Table 3). On the contrary, the frequency of haplotype T-T-T, which included the major alleles of rs5766741, rs5767700, and rs1800234, was significantly lower in Sherpa highlanders (0.73) than non-Sherpa lowlanders (0.85, *p* = 0.003), with an OR of 0.49 (95% CI = 0.31–0.49) (Table 3). On the contrary, the block 1 included rs5769178 and rs881740 of two SNPs that covered 6 kb length of the gene. The frequencies of the haplotypes in block 1 (A-A and C-G) were not significantly different between the two study groups (Table 3).

**Table 3.** The Haplotypes of the Seven Single-Nucleotide Polymorphisms in the Gene Encodes Peroxisome Proliferator-Activated Receptor-Alpha in Sherpa Highlanders and Non-Sherpa Lowlanders

Haplotypes			Sherpas (*n* = 105)	Non-Sherpas (*n* = 111)	*p*	OR (95% CI)
			Frequency	Frequency		
Block 1 (6 kb)						
rs5769178 (A > C)	rs881740^a^ (A > G)					
A	A		0.84	0.87	0.3066	0.75 (0.44–1.29)
C	G		0.16	0.11	0.0796	1.65 (0.94–2.92)
Block 2 (10 kb)						
**rs5766741** ^a^ **(T > C)**	**rs5767700 (T > C)**	**rs1800234** ^b^ **(T > C)**				
**C**	**C**	**C**	**0.11**	**0.03**	**0.0016**	**3.74 (1.57–8.92)**
**T**	**T**	**T**	**0.73**	**0.85**	**0.0030**	**0.49 (0.31–0.49)**
C	C	T	0.13	0.10	0.2816	1.38 (0.76–2.51)
T	C	T	0.03	0.02	0.4346	1.76 (0.42–7.48)

Bold indicates data that are significantly different between the two populations. The SNP variant is indicated as the major allele > the minor allele. The *p* values were calculated using the chi-square test (2 × 2 contingency table).

^a^ eQTLs, the genomic loci that contribute to variations in the mRNA expression levels of the *PPARA*.

^b^ The nonsynonymous substitution V227A in the *PPARA*.

*PPARA*, the gene encoding peroxisome proliferator-activated receptor-alpha.

## Discussion

Consistent with the literature about the physiology of Sherpas (Gilbert-Kawai et al., 2014), the present data concerning the high-altitude residence, remarkable mountaineering ability, absence of symptoms of CMS, and relatively high SpO_2_ at high altitude of Sherpa highlanders verified again that the Sherpas is a distinctive population adapted to high altitude (Gilbert-Kawai et al., 2014). They are significantly different from the populations who originated from the sea level or low altitude in respect of the physiological tolerance to high-altitude hypoxia.

The results of allele discrimination suggested that the population stratification was minor regarding these seven SNPs between the Sherpa highlanders and non-Sherpa lowlanders (Supplementary Table S2). Therefore, we analyzed the genetic information of the seven SNPs in the Sherpa highlanders with reference to that in the non-Sherpa lowlanders. We believe that the comparisons are reasonable and reliable regarding these genetic polymorphisms between the two study groups.

The main findings of this study were as follows: (1) that the frequencies of the minor alleles of rs4253712, rs5766741, rs5767700, and rs1800234 were significantly overrepresented in the Sherpa highlanders in comparison with non-Sherpa lowlanders and (2) that the frequency of the haplotype that included the minor alleles of rs5766741, rs5767700, and rs1800234 was significantly overrepresented in the Sherpa highlanders in comparison with non-Sherpa lowlanders. The strengths of the minor alleles and the haplotype constructed with these minor alleles showed powerful positive ORs in Sherpa highlanders. These results strongly suggest that the genetic variations of these SNPs in the *PPARA* are significantly predominant in Sherpa highlanders, probably because of natural selection by exposure to the high-altitude environment for hundreds of generations.

Among the significant SNPs of rs4253712, rs5766741, rs5767700, and rs1800234, two SNPs (rs4253712 and rs5766741) are eQTL loci and one (rs1800234) is nonsynonymous SNP in the *PPARA*. Moreover, the significant haplotype was linked with nonsynonymous rs1800234 and eQTL locus rs5766741. These eQTL loci were reported in contribution to variations in the mRNA expression levels of the *PPARA* in an Asian population (Narahara et al., 2014; Higasa et al., 2016). These significant eQTL loci will be further verified concerning their impact on the variations in mRNA expression levels of the *PPARA* in Sherpa highlanders in future study. The nonsynonymous rs1800234 substitutes valine to alanine at codon 227 in the PPAR-α receptor. It is located in the hinge region between the DNA-binding and the ligand-binding domains of the *PPARA* gene. It plays a critical role in the functional alteration of the PPAR-α receptor and is associated with variations in metabolic levels (Yamakawa-Kobayashi et al., 2002; Naito et al., 2006).

Simonson et al. (2010) identified the genetic association of the *PPARA* with Tibetan highlanders in the metabolic adaptation to high-altitude hypoxia. Ge et al. (2012, 2015) also reported that the selected *PPARA* haplotype was correlated with serum free fatty acid levels in Tibetan highlanders. Recently, Horscroft et al. (2017) convincingly demonstrated that the constitutional genetic variants of the *PPARA* were underlaid with Sherpa highlanders for metabolic adaptation to high altitude. We speculate that this kind of genetic variations of the *PPARA* in Sherpa highlanders might involve with the establishment of an appropriate level of metabolism at high altitude for adaptation to hypoxia environment. Under the high-altitude hypoxic circumstance, the energy balance can be achieved despite low oxygen levels by preferentially utilizing glucose or glycogen rather than free fatty acids or lipids in Sherpa highlanders (Morpurgo et al., 1976; Holden et al., 1995; Hochachka et al., 1996a).

One limitation of this study was that there were no data for the metabolic phenotypes in the study groups, such as the levels of triglycerides, free fatty acids, β-hydroxybutyrate, and lactate, because of inadequate blood samples. Another limitation was the relatively small sample sizes of the study groups, which might diminish the statistical power. Nevertheless, our samples size was necessary to find the OR of 3.0 statistical significance (two-sided test) in a case–control study for a power of 80%. In addition, the *p* values were corrected for multiple hypothesis tests in the adjustment of the probability of resulting in false positive. Advanced study is needed to minimize these limitations to confirm the presence of naturally selected haplotypes or other significant SNPs in the *PPARA* gene in Sherpa highlanders for elucidating the metabolic adaptation to high-altitude hypoxia.

In conclusion, this study demonstrates that the *PPARA* is one of the effective functional genes in association with the metabolic adaptation to high-altitude hypoxia in Sherpa highlanders.

## Supplementary Material

Supplemental data
