# Supplementary material for: The Contribution of Genetic Variants of the Peroxisome Proliferator-Activated Receptor-Alpha Gene to High-Altitude Hypoxia Adaptation in Sherpa Highlanders
Source: High Alt Med Biol. 2023 Sep 12;24(3):186–92. doi: 10.1089/ham.2018.0052 (PMC10516232; doi:10.1089/ham.2018.0052)
Supplement: Supplemental data [file Supp_Data.pdf]

Supplementary Data

Supplementary Table S1. The Genetic Distances (F_{ST}) of the Seven Single-Nucleotide Polymorphisms in the Gene Encodes Peroxisome Proliferator-Activated Receptor-Alpha Between South Asians and East Asians According to 1000 Genomes Project Genotype Data

SNPs	F_{ST} *between South and East Asians*[a]
rs135547	0.05
rs5769178	0.005
rs881740[b]	0.005
rs4253712[b]	0.0005
rs5766741[b]	0.0003
rs5767700	0.006
rs1800234[c]	0.02

http://grch37.ensembl.org/Homo_sapiens/Info/Index

[a]F_{ST} of the seven SNPs was measured with Genepop software package (Guo and Thompson 1992).

[b]eQTLs, the genomic loci those contribute to variations in the mRNA expression levels of the *PPARA*.

[c]The nonsynonymous substitution V227A in the PPAR-α receptor.

eQTL, expression quantitative trait loci; *PPARA*, the gene encodes peroxisome proliferator-activated receptor-alpha; PPAR-α, the gene encoding peroxisome proliferator-activated receptor-alpha; SNP, single-nucleotide polymorphism.

Supplementary Reference

Guo SW, and Thompson EA. (1992). Performing the exact test of Hardy-Weinberg proportion for multiple alleles. Biometrics 48:361–372.

Supplementary Table S2. The Genetic Distances of the Seven Single-Nucleotide Polymorphisms in the Gene Encodes Peroxisome Proliferator-Activated Receptor-Alpha in Sherpa Highlanders Versus Non-Sherpa Lowlanders

SNPs	*Fixation index between Sherpa highlanders and non-Sherpa lowlanders*[a]
rs135547	0.0704
rs5769178	0.0103
rs881740[b]	0.0124
rs4253712[b]	0.0695
rs5766741[b]	0.0823
rs5767700	0.0705
rs1800234[c]	0.0883

[a]F_{ST} of the seven SNPs was measured with Genepop software package (Guo and Thompson 1992).

[b]eQTLs, the genomic loci those contribute to variations in the mRNA expression levels of the *PPARA*.

[c]The nonsynonymous substitution V227A in the PPAR-α receptor.
